# Community-based initiatives improving critical health literacy: a systematic review and meta-synthesis of qualitative evidence

**DOI:** 10.1186/s12889-017-4570-7

**Published:** 2017-07-20

**Authors:** Liesbeth de Wit, Christine Fenenga, Cinzia Giammarchi, Lucia di Furia, Inge Hutter, Andrea de Winter, Louise Meijering

**Affiliations:** 1 University of Groningen, Population Research Centre / Urban and Regional Studies Institute, Landleven 1, P.O. Box 800, 9700 AV Groningen, The Netherlands; 2 Amsterdam Institute for Global Health and Development, Trinity Building C, 3rd Floor Pietersbergweg 17, P.O. Box 22700, 1100 DE Amsterdam, The Netherlands; 3 Agenzia Regionale Sanitaria (The Regional Agency for Health), Marche Region, Via Gentile da Fabriano n.3 nel palazzo Rossini, 60125 Ancona, Italy; 4 International Institute of Social Studies, The Hague, of Erasmus University Rotterdam, The Netherlands, Kortenaerkade 12, 2518 AX The Hague, The Netherlands; 5 Department of Health Sciences, University of Groningen, University Medical Center Groningen, Postbus 196, 9700 AD, Groningen, The Netherlands

**Keywords:** Systematic review, Meta-synthesis, Qualitative data analysis, Critical health literacy, Community-based initiatives, Older adults, Communities, Social and cultural context

## Abstract

**Background:**

Critical health literacy enables older adults to make informed health decisions and take actions for the health and wellbeing of themselves and their community, within their own social and cultural context. A community-based approach has the potential to improve the critical health literacy of older adults and their communities. However, it is not clear how such initiatives consider critical health literacy. Therefore, this study explored how community-based initiatives address the critical health literacy of older adults and their communities.

**Methods:**

A systematic literature search was conducted. Two reviewers independently screened titles and abstracts, as well as the quality of the methodological and community-based elements of the studies. In addition, a meta-synthesis was carried out, consisting of a qualitative text analysis of the results sections of the 23 included studies.

**Results:**

We identified two main themes, which are practices that contribute to the critical health literacy of older adults as well as their communities: 1) collaborative learning, and 2) social support. In these practices we identified reciprocity as a key characteristic of both co-learning and social support.

**Conclusions:**

This study provides the first overview of community-based initiatives that implicitly address the critical health literacy of older adults and their community. Our results demonstrate that in the context of one’s own life collaborative learning and social support could contribute to people’s understanding and ability to judge, sift and use health information. We therefore suggest to add these two practices to the definition of critical health literacy.

**Electronic supplementary material:**

The online version of this article (doi:10.1186/s12889-017-4570-7) contains supplementary material, which is available to authorized users.

## Background

In many countries there are wide disparities in the health of different social groups [1]. An important and growing group of people at risk of poor health are older adults [2]. In the debate on tackling health inequalities, health literacy (HL) has been acknowledged by the World Health Organization as a key determinant of health and wellbeing [3]. Health literacy concerns the knowledge and competences to access, appraise and apply health information in order to make health decisions [4]. Research has shown that by enabling older adults to find, access and use health information, their health can improve [5, 6]. However, the majority of HL research approaches the functional health literacy of individuals, without considering their social and cultural context, as well as actions and abilities at the group level. The critical health literacy (CHL) concept encompasses the context in which people live, and entails: “the understanding and ability to judge, sift and use information provided in the context of one’s own life” ([7] p.294). Thus, CHL has the potential to address health inequalities in a more comprehensive way than functional approaches to health literacy. Besides its emphasis on context, CHL refers to abilities and actions at both the individual and group level [8]. However, many researchers have articulated a lack of initiatives to improve CHL and reduce health inequalities at the group level, such as community-based initiatives [9–11]. A community-based approach to CHL has the potential to let community members participate in exchanging and co-creating health knowledge, within their own context. Currently, there is no overview of such initiatives.

### Critical health literacy

Several researchers in the field of public health and health promotion explored the concept of CHL [12–15]. Overall, CHL covers three main, overlapping, areas: 1) individual level abilities and actions to manage health, 2) individual level abilities and actions on the social determinants of health, 3) group-level abilities and actions on the social determinants of health to manage health.

The first CHL area involves critical thinking, informed decision making, and exerting control over health and disease. It involves people’s cognitive skills that enable someone to contextualize health information and apply it to one’s personal situation and context, in order to make an informed decision that benefits health and wellbeing. This area of CHL can be viewed as an asset, supporting people to engage with health information and the health care system, and exert greater control over people’s health in the context of their life.

The second area of CHL acknowledges the importance of existing structural factors that indirectly influence someone’s health and wellbeing, comprising social and community networks, living and working conditions, and socio-economic, cultural and environmental conditions [16]. This area of CHL is strongly influenced by the demands and complexities of people’s individual and social contexts. Determinants that have large impact on people’s health and wellbeing are, for instance, health care systems, and health and social policies. CHL encompasses the empowerment of people to challenge and take actions regarding these determinants of health and wellbeing. Mogford et al. provide an example of a school-based initiative, which involves a health education curriculum that aims to teach students CHL skills and develop and implement actions on the social determinants of health [10].

The third area of CHL is about people engaging in collective activities regarding health issues, such as shared decision making. According to Chinn (2011), CHL competences needed for collective actions involve, for example, skills in working groups and knowledge of the local community [14]. Mogford et al. (2011) view this CHL area as a pre-condition for taking health actions in the community and developing public policy [10].

### Community-based approaches

A community can be defined as a group of people having a shared identity, based on specific characteristics, for example their culture [17]. Community-based approaches are often labelled as Community-based Participatory Research and Participatory Action Research initiatives, and are often applied in the field of health [17, 18]. Key to community-based approaches is that they bring people together, offer the opportunity to share knowledge and experiences, and create common understandings. Such approaches aim to empower participants and their communities through their roles as active agents throughout the whole process. Furthermore, a community-based approach focusses on building strengths and resources within communities as a unit, and forging equitable partnerships to foster capacity building for the mutual benefit of all partners [17]. Finally, community-based approaches emphasize the development of sustainable actions at the individual and community level [18].

A community-based approach is well suited for CHL initiatives for two reasons. First, they both focus on empowering individuals by gaining health knowledge and carrying out actions for health and wellbeing. People are stimulated to critically reflect on their own knowledge and experiences regarding health and social issues, and to act upon it. Second, both a community-based approach and CHL initiative take place at the community level, incorporating people’s social and cultural context, allowing for sharing health information, and enabling collective understandings and actions.

In this study we conducted a systematic review and meta-synthesis of qualitative evidence, with the aim of exploring how community-based initiatives address the CHL of older adults and their communities.

## Methods

### Search strategy and selection of studies

Searches were carried out in five electronic databases: PubMed, Embase, Web of Knowledge, PsycINFO and CINAHL, as well as the databases of the World Health Organization (WHOLIS) and FP7 IROHLA program. The latter concerned several databases of health literacy interventions with older adults in Europe, generated through multiple systematic reviews. Besides, reference lists of the articles that were included for data-extraction were searched. Different search strings were developed and tested and led to a search strategy, which encompassed terms related to ‘community-based initiatives’ and ‘older adults’ (see Additional file 1). The term ‘critical health literacy’ as one term was tested for incorporation in the search strategy; however, no results were found in combination with the other search terms. Adding a search string containing various single terms related to ‘critical health literacy’, such as informed decision making and empowerment, was considered. However, as CHL is a broad concept, we would have missed many relevant articles with this strategy. Therefore, the CHL concept was not included in the search strings, but rather used as an inclusion criteria. Two reviewers screened the studies for CHL using the definition of the CHL concept as presented in the introduction of this article [7]. No restrictions were imposed with regard to year of publication. Inclusion criteria were: qualitative studies with a participatory methodology, with older adults aged 50 years and older, carried out in a community setting, and concerning critical health literacy. Studies written in another language than English, Dutch or Italian were excluded. Each title and abstract were screened independently by two reviewers. The team of reviewers consisted of CF, LM and CG. All papers were screened by two reviewers whereby differences were resolved by discussion. When differences in opinion persisted, a third reviewer was involved. All phases of this review, i.e. developing the search strategy, selecting studies, assessing quality and analyzing data, were pilot tested. Besides, regular group discussions with the review team took place throughout the review process. In accordance with good practice for systematic reviews, the study protocol was based on the Cochrane handbook for Systematic Reviews of Interventions [19] and PRISMA guidelines [20] (see Additional file 2).

### Quality assessment, data extraction and data synthesis

Full text articles of the studies that met the inclusion criteria were retrieved and evaluated for their quality. We assessed both the methodological quality and the quality of the community-based approach. To achieve this, we combined the CASP tool for critical appraisal of qualitative research [21], and a tool for quality rating of community-based studies, developed by Viswanathan et al. [22] (see Additional file 3). The quality of the study aim and methodology was assessed first, by one reviewer (LM), as this was relatively straightforward. In case of doubt a second reviewer (CF) was consulted. Subsequently, the quality of community-based elements was assessed by two reviewers (CF and CG) independently, and disagreements were resolved by discussion or a third reviewer (LM) if necessary. Results of the quality assessment are available from the corresponding author on request. Three members of the review team (CF, CG and LW) were involved in extracting characteristics from the included studies (see Table 1).

**Table 1:** Characteristics of the 23 included studies

Ref	Study (year)	Study aim	Target population; study setting	Actors participating in the study	Data collection methods	Pre-dominant theme	
						co-learning	social support
General older adult population							
25	Hamrosi Bpharm et al. (2014)	To explore needs and expectations regarding written information of medicines, and to determine related barriers and facilitators	People who had taken at least one medicine prescription in the last 12 months, aged 21–80; New South Wales, Australia	priority community, researchers	focus groups		X
26	O’Brien et al. (2011)	To explore the concept of elder abuse by engaging with older people living in the community	Older adults, aged 65–89; Ireland and Northern Ireland	older adults, practitioner, community worker, researchers	focus groups		X
27	Punnaraj et al. (2010)	To develop a community health care model for providing care to older adults	Older adults, aged 60+; Northeast Thailand	older adults, family members, community leaders, nurses, health care volunteers, local government officials, researchers	interviews, participatory observations, focus groups	X	X
28	Sanders et al. (2006)	To develop and evaluate an education programme to help older people find out about treatment and care choices at the end of life	Older adults;Sheffield, UK	voluntary community advisors representing older adults, community members, researchers	workshops	X	
29	Valokivi (2004)	To explore citizenship in the local health care and social service system	Older adults who received care, aged 80–94; rural middle sized municipality in Finland	older adults, informal caregivers(relatives), researcher	semi-structured interviews		X
30	Ward (2014)	To explore how older people learn to sustain their own and others’ well-being	Older adults, aged 60–97; UK	older adults, voluntary sector manager, researchers	interviews, focus groups		X
Older adults with a (risk of) disease							
31	Adili et al. (2013)	To explore the ways in which women learned to live with type 2 diabetes	Women with diabetes, aged 55–82; Newcastle, Australia	older women, family members, researcher	Interviews, informal conversations, discussion groups	X	X
32	Espenschied et al. (2012)	To explore the needs for post genetic-cancer-risk-assessment of patients with increased risk for breast and/or ovarian cancer	Women with increased cancer risk, aged 26–74; Hope, USA	older women, family members, professionals, field experts, clinical researchers	Interactive conference	X	X
33	Evans et al. (2007)	To identify key messages about pre-diabetes, and to develop an educational toolkit to address the information needs of patients and health care professionals	Patients with (pre-) diabetes, aged 48–79, and health professionals; urban and rural areas in UK	(pre-) diabetes patients, health professionals, diabetes experts, researchers	focus groups, interviews, videotaped consultations	X	X
Ethnic minorities and migrants							
34	Andrews et al. (2007)	To develop a culturally sensitive smoking cessation intervention with public housing neighborhoods	African American residents of public housing neighborhoods; Augusta-Richmond county, Georgia, USA	neighborhood residents, community health workers, community advisory board, researchers	neighborhood surveys, focus groups, interviews	X	
35	Balbale et al. (2014)	To develop culturally tailored health messages by using visual and participatory action techniques	Mexican females, aged 65+; Chicago, USA	Mexican older women, researchers	photo-elicitation, in-depth interviews, focus groups	X	X
36	Boise et al. (2013)	To explore perceptions regarding health needs and barriers to health care	African immigrants and refugees, aged 14–67; Portland, USA	African immigrants and refugees, family members, service providers, researchers	informal conversations, community meeting, house meetings		X
37	Collier et al. (2012)	To identify mental health needs and ideal service delivery systems of/for a Hmong community	Laos Hmong community, aged 18–70; Eau Claire, USA	Hmong community members, Hmong professionals, community service providers, researchers	focus groups, key informant interviews		X
38	Cusack et al. (2013)	To explore knowledge, use and experiences of over-the-counter analgesics	Aboriginal community, aged 20–80; north-west Adelaide, Australia	Aboriginal community, Aboriginal Elder, researchers	focus groups, interviews	X	X
39	Holkup et al. (2007)	To prevent elder abuse by an elder focused, family centered, community-based conference	Native American older adults; northwestern community in USA	older adults, family members, community members, spiritual leader, health and social service providers, researchers	Family Care Conference	X	
40	Petrucka et al. (2007)	To explore capacity building and cultural competency in health professional education and health-care delivery	Two Saskatchewan Aboriginal communities; southern Saskatchewan, Canada	community members, Aboriginal Elders, researchers	hearing circles	X	
41	Zanjani and Rowles (2012)	To increase knowledge on mental health and aging, and to explore culturally appropriateness of interventions on mental health-related topics	Older adults, aged 65+; rural Appalachian-Kentucky, USA	community members, professional community liaisons, researchers	focus groups	X	X
Ethnic minorities and migrants with a disease							
42	Braun et al. (2002)	To examine cancer experiences, including the impact of fatalistic attitudes, access to healthcare, and culturally linked values	Native Hawaiians who survived cancer, aged 36–86; Hawai, USA	cancer patients, native Hawaiian social worker, native Hawaiian co-investigator, native Hawaiian clinicians, researchers	focus groups		X
43	Carlson et al. (2006)	To explore how communities use and value the internet and libraries for health information	African Americans with and without diabetes, aged 60+; Charleston and Georgetown counties, USA	community members, faith community, public librarians, diabetes advocacy group, rural community center staff, information technology community members	survey, focus groups	X	X
44	Eggly et al. (2013)	To develop a communication tool to address racial disparities in cancer care	Black African-American cancer patients, aged 40+; southeast Michigan, USA	cancer patients, community advisory committee, representatives of community health organizations, oncologists, researchers	semi-structured interviews	X	X
45	Grigg-Saito et al. (2008)	To eliminate health disparities in cardiovascular disease and diabetes by increasing knowledge, access to health care and awareness of health care beliefs	Cambodian older adult refugees, aged 50+; Lowell, USA	Cambodian community, community leaders and elders, health providers, human services staff, government agency staff, researchers	surveys, focus groups	X	
46	Kieffer et al. (2004)	To reduce disparities in diabetes by reducing the prevalence and impact of diabetes and its risk factors	African-American and Latino community members, aged 8–76; eastside and southwest Detroit, USA	community members, family members, community-based organizations staff, policy makers, health system developers, community health center staff, researchers	focus groups	X	X
47	Pierre-Louis et al. (2011)	To understand experiences of people with diabetes and to envision new patterns of health	African American women with diabetes, aged 35–68; urban community, USA	African American women with diabetes, nursing students, researcher	Interviews		X

Various methods for data synthesis are used in qualitative systematic reviews [23]. In this study, we integrated the findings from individual studies through performing a qualitative text analysis on the results sections of the individual articles [24]. Two researchers (LW and CF) coded a subset of the data in parallel, each drafted a code tree, discussed these and developed one main code tree that formed the basis for further coding. The code tree was continuously adapted throughout the coding process based on emerging findings. In iterative cycles, the codes were compared, categorized under specific subthemes, and conceptualized under the two main themes presented in the findings. The analysis was continuously discussed among the authors, LW and LM in particular, to ensure the credibility of the findings.

## Results

**Fig. 1:**
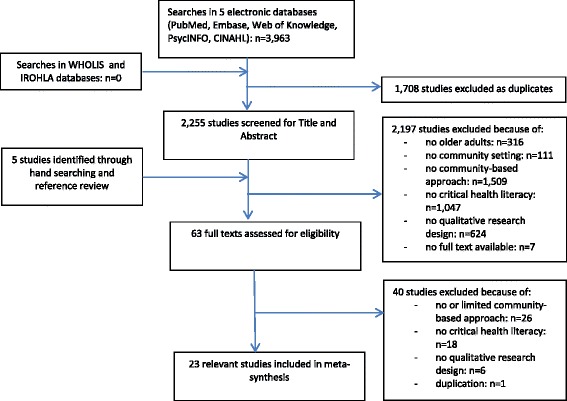
Flowchart of included studies

In total 3963 studies were identified, of which 1708 were excluded as duplicates. 2255 Articles were screened for title and abstract. The review team initially disagreed about 293 studies, which were discussed. Finally, 2197 articles were excluded. A second screening on exclusion criteria and a quality assessment was done of 63 full texts, 40 were excluded, resulting in 23 articles for data synthesis (see Fig. 1).

### Study characteristics

Table 1 presents the characteristics of the 23 studies that were synthesized in this review. Nine studies concerned older adults in general, with or without a chronic disease, who lived in a western culture [25–33]. Thirteen studies concerned minority populations living in a western culture [34–47]. One study [27] was carried out with older adults in a non-western culture. Nine studies aimed to describe the process of the community-based approach [27, 28, 32–35, 39, 44, 45]. All studies targeted CHL implicitly. Some examined CHL by exploring health knowledge and abilities of older adults in their community context [25, 38, 41, 43]. Most studies addressed CHL through factors that indirectly influenced older adults’ health and wellbeing, including personal and lifestyle factors, cultural conditions, and the health care system [26, 27, 29–37, 39–47]. Three studies [30, 43, 46] addressed CHL through focusing on collective actions. Furthermore, almost all studies, except for two studies [25, 35], involved other actors such as family members, people with particular knowledge of the community, and/or health professionals or service providers.

### Overall findings from the data synthesis

We identified two main themes in the data, which are practices that contribute to the critical health literacy of older adults and their communities: 1) collaborative learning, and 2) social support. Through co-learning and the exchange of social support, older adults and their community gain health knowledge and utilize care. In contrast, sometimes co-learning and social support served as a potential barrier to developing CHL. In each of these practices, reciprocity was identified as a key characteristic.

#### Collaborative learning

In the studies we analyzed, collaborative learning, or co-learning, was expressed as a way of gaining health knowledge within the community through sharing knowledge. Co-learning involved situations where older adults interacted and exchanged health knowledge with family, community members or peers, as well as health care professionals, in order to learn from each other.

##### Family and community

In many studies older adults shared their knowledge, experiences and wisdom of health issues and health information with younger generations in their family and community [27, 34, 35, 38–41, 43, 45, 46]. In this way, family and community gained health knowledge from older adults: *“All of them wanted to remain contributing members of their family unit and community, because they viewed themselves as having knowledge and experiences, due to their extended years of life, which others may not have”* [27]. Social and cultural norms in the community influenced how the knowledge that older adults gained throughout their lives was valued by and brought back into the community. Almost all studies involved communities that were founded on social and cultural values of sharing knowledge. In such communities older adults often played a special role as ‘health knowledge providers’, as they were highly valued for their knowledge, experiences and wisdom. For example, in some Aboriginal communities [38, 40] ‘trusted elders’ assisted community health workers with advising community members about health issues, health information or medication: *“Participants reported that Aboriginal people seek guidance from their Elders because of their wisdom and life experience; the Elder is still here and they have the wisdom and experience of living and that is why we believe them.”* [38]

##### Peers

In several studies older adults expressed the importance of learning from each other about health and disease [27, 28, 31–33, 44–46]. This type of co-learning was particularly discussed by older adults sharing a (high risk of) disease, such as diabetes or cancer, and mainly concerned co-learning through support groups. Older adults learned from each other through sharing experiences, and exchanging information regarding treatment and coping with their diseases. Experiences and knowledge concerned issues on how to manage information, disease, treatment and consultations with the doctor: *“What would help to improve the experience of getting overwhelmed by information provided during genetic counseling is to have “an opportunity to talk with other women who were gene positive… to get information from them, maybe their own experience””.* [32]

However, sometimes older adults did not have access to support groups, and could therefore not learn from others in this way [27, 42]. This concerned older adults living in rural areas [42], and a community where facilities for meetings were not available [27].

##### Health care professionals

Several studies addressed co-learning between older adults and health care professionals, such as doctors and formal caretakers [25, 36, 40, 42, 44]. Co-learning between older adults and professionals particularly concerned the sharing of health knowledge, for example through asking questions to the doctor, or sharing decision making. A relationship of trust and empathy between older adult and health care professional was essential for co-learning [25, 36, 40]. When older adults and health care professionals had different cultural backgrounds this relationship was even more challenging [36]. Speaking a different language could further increase communication difficulties. In some cultures, exchanging knowledge between different actors was embedded, which is illustrated by the following quotation: *“A theme encapsulated in the phrase “the teacher must be taught/the caregiver must receive care” intimated that there is reciprocity in all aspects of the relationship such that those providing knowledge must also be receiving knowledge. The participants expressed the view that although professionals are recognized as knowledgeable, they have a unique opportunity to experience complementarity with Aboriginal/traditional ways of knowing.”* [40] In this study, health care professionals were taught Aboriginal ways of caring by the patient and his/her family, whereas they, in turn learned about western medical practices from the health professionals.

#### Social support

We discuss social support as older adults gave it to and received it from their community, family, peers and health care professionals.

##### Community

In several studies, community members such as community leaders, community workers or neighbors, provided older adults with social support [26, 27, 29, 30, 34, 46, 47]. Here, community-based knowledge and practices contributed to the health knowledge, skills, health and wellbeing of older adults. For example, community members negotiated and arranged care for older adults: *“The interviewee’s most important caregiver and advocate is the lady next door. She takes care of matters related to his everyday care and visits him on a daily basis. She is the first person whom he contacts concerning the need for help and any changes in it.”* [29]

In some studies, older adults provided social support to their community members [27, 38, 40, 41]. This occurred most often in ethnic minority and migrant communities, where older adults carried out leadership roles, chaired health care volunteers, or provided health and medication information to community members: *“Many [older adults] continued to contribute significantly to their community as workers or cultural advisors in a variety of settings including health, welfare, education, courts and correctional services.”* [38]

Three studies underlined the reciprocity of social support in the community. Some older adults wanted to give something back to the community, for example by doing voluntary work, because they had received care earlier in their life [30]. At the same time, several older adults expected something back from the community in return for what they had done for the community [29, 47]. These expectations were not always fulfilled: *“They described expending a large amount of energy caring for other people, but they had no one to care for them. These women had few community supports … None of the women spoke of having people available to provide them with the same quality of care, support, and nurturance they provided to others.”* [47]

##### Family

In several studies family members provided older adults with social support [26, 27, 29–31, 34, 42–44, 46, 47]. Children and spouses assisted with health information, arranging care, transport to health services, doctor’s visits, medication, monitoring illness, safety issues or mental health issues. A recurring topic was the role of children in making decisions with their parents [26, 27, 29, 46]: *“The aged woman was active in making an assessment of her need and authorized her family member to seek for it. Her social network was there to support her and act on her behalf.”* [29] In this example the older woman and her son shared decision making about the health care that she needed after leaving the hospital. However, in some studies older adults expressed the need for family support where this was not provided to them, for instance because they did not have family members to rely on, their family was overburdened, or family habits did not allow social support [26, 46, 47]. In the following citation, the lack of social support in food choices in the family was a barrier to healthy eating for an older man with diabetes: *“Johnny got diabetes but he’s gonna have to eat like the rest of us [his family]”*. [46]

Some studies particularly discussed older adults giving social support to their families [27, 30, 35]. These older adults cared for their grandchildren or other family members, mostly because they wanted to keep contributing to the functioning of their family: *“During times of family illnesses, these elders served as health care providers and often took ill family members to the physician, as well as helped with the family’s activities of daily living.”* [27]

Several studies described the reciprocity of social support within families [26, 27, 30, 46, 47]: *“The care that Elsie received from her family felt less like a burden as she felt she still had an important role within her family as mother and grandmother.”* [30] In this example the older woman and her family members contributed to each other’s health knowledge, experiences and skills.

##### Peers

In contact with peers, the reciprocal nature of social support was important [26, 29–31, 34, 43, 47]. Community groups and close friends were important for older adults for exchanging personal health experiences and information: *“Friends were another important source of support to older people and in some instances were thought to understand the older person’s life better than the older person’s children…”*. [26] However, a social network of close friends is not self-evident, and in later life in particular subject to decline when close friends pass away.

##### Health care professionals and services

In contrast to the more informal social support provided by the community, family and peers, older adults received more formal social support from health care professionals. In many studies older adults expressed what social support they expected to receive from health care professionals and services [25, 26, 29, 30, 33, 37, 40, 43, 44, 47]. Care workers were expected to provide adequate care, treat people with respect, show empathy, share knowledge of the health care system, and co-operate with older adults’ relatives. Perceived responsibilities of doctors included speaking to older adults, providing medical information, seeking people’s views, and discussing treatment options. Older adults with a disease particularly talked about the role of their medical specialists, which concerned providing information about their disease and disease management, discussing treatment options, and knowing how to deal with sensitive information, as expressed by a women with cancer: *“One question I don’t want to know is how long do I have. I don’t want to know. I think if a person wants to know, they should ask, I don’t think the doctor should tell them.”* [44]

Although older adults indicated that it was their own responsibility to ask for health information, take part in decision making about care and treatment, and make lifestyle changes, in many studies older adults articulated barriers in taking the responsibility for their own health [25, 26, 29, 32, 36, 37, 40, 42, 44, 46, 47]. Often, they felt uncomfortable in consultations with doctors and specialists, which restrained them from speaking out. Different social and cultural norms of the older adult and health care professional further contributed to the older adult’s feeling of discomfort: *“The value of truth was a recurrent theme relating to openness, transparency, honesty, and trust. Participants perceived a high level of distrust and misinformation by both Aboriginal and non-Aboriginal partners in the current health-care environment.”* [40] Often, social and cultural norms of ethnic minorities and migrants concerned communication style and respectfulness towards each other: *“Interviewees [Laos community in USA] reported that a confrontational style does not work well with Hmong; it was seen as disrespectful, especially if there is an age difference between the patient and the provider”.* [37]

## Discussion

The findings of this study show how community-based initiatives have the potential to address the CHL of older adults and their communities, and in so doing provide insight into the concept of CHL at the community level. We identified two practices that contribute to the CHL of older adults and the community: collaborative learning and social support. Collaborative learning and social support with reciprocity could empower older adults to access health information, to make informed decisions, and to arrange care in their community context.

The findings of this study demonstrate how *co-learning* can contribute to the CHL of older adults, the community and health care professionals. Co-learning can improve the CHL of older adults through support groups where older adults with a disease learn from each other. The CHL of communities can improve through co-learning when older adults share their health knowledge gained throughout their lives with the community. Furthermore, the CHL of health care professionals and older adults could improve through co-learning, by sharing knowledge about culture, disease, health care and treatment.

Although we did not find other studies that explicitly connect co-learning to CHL, there are studies that linked the broader concepts of literacy and learning. Xie argued that co-learning can be a useful method for improving older adult’s e-health literacy [48]. Besides, Gokhale emphasized the importance of co-learning for critical thinking, an important part of CHL [49]. He argued that individuals are able to retain more information and achieve higher levels of learning when they work in a group rather than individually. This concerned both facilitators and receivers of knowledge. In addition, Sykes et al. explicitly linked learning to CHL: “CHL is a learned and movable state that may change with time or circumstances of people’s lives” [15, p.6]. The link between community settings and co-learning has been acknowledged in many studies. Israel et al. argued that community-based participatory research fosters co-learning among its participants, since it recognizes that all participants bring in diverse perspectives, experiences, knowledge, and skills [50]. This is supported by other authors, such as Freire [51].

Our findings demonstrate how *social support* can contribute to the CHL of older adults, the community and health care professionals. In our findings, social support could be linked to four types of social support identified by House: emotional (e.g. sharing experiences), instrumental (e.g. tangible aid), informational (e.g. advice and information), and appraisal (e.g. information for self-evaluation) [52]. Our findings suggest that emotional support was most often provided by community members and family, and could improve older adult’s CHL through sharing health experiences and gaining health knowledge. Besides, older adults reciprocated emotional support with their peers, contributing to their CHL. Instrumental support was most often provided by community and family members who contributed to the CHL of older adults through enabling access to health services and care. Besides, older adults gave instrumental support to their family, contributing to the family’s CHL. Informational support was most often provided by family members, and could contribute to the CHL of older adults through sharing health information, gaining health knowledge, and improving abilities and skills. Besides, older adults reciprocated informational support with their peers, contributing to their CHL. Appraisal support was provided less often, but sometimes by family and health care professionals, and could contribute to the CHL of older adults through shared decision making. Furthermore, our findings show that older adults expected to receive all types of social support from health care professionals and services. At the same time, older adults perceived a lack of emotional support from health professionals, specifically trust, empathy and understanding of a patient’s cultural background. The perceived lack of emotional support forms a barrier for older adults to develop CHL skills, such as accessing and judging health information.

According to our knowledge there is no other literature that illustrates the contribution of social support to CHL. However, two studies of Lee et al. investigated social support in relation to the general concept of health literacy [53], and the functional health literacy domain [54]. In their first study, Lee et al. argued that social support can improve a person’s ability to acquire and understand medical information and to negotiate the health care system, which was confirmed by our review [53]. In a follow-up study, Lee et al. found that social support has a more positive impact on the health of older adults with high functional health literacy [54]. Our review did not confirm these findings when exploring the CHL of older adults, since we took the wider social and cultural aspects of communities into account in exploring social support.

### Methodological considerations

A main strength of this review is its comprehensive approach to exploring the CHL of older adults and their communities in community-based initiatives. In addition, the method of systematic review and meta-synthesis made it possible to provide a broader understanding of how CHL could be strengthened though community-based initiatives. A weakness of our review is that we mostly draw on English written evidence. In spite of this, the included studies involved a diversity of communities. Another potential weakness is that most of the included studies were located in western English-speaking countries. We had expected to find more studies in non-western settings, since community-based initiatives have a long history there. A reason for the lack of these studies in our review could be our focus on older adults. Because of the relatively low proportion of older adults in non-western populations, older adults are less often studied.

## Conclusion

This systematic review and meta-synthesis made the CHL of older adults and their community visible in community-based initiatives. It demonstrates that older adults gain much health knowledge within the community through collaborative learning and social support. Therefore, we conclude that these two practices should be added to the concept of CHL. Based on our review, we propose the following definition of CHL: the understanding and ability to judge, sift and use health information provided in the context of one’s life, through individual and community practices, such as co-learning and social support. This definition explicitly encompasses the community, as well as the role of co-learning and social support.

When looking at the implication of our findings, we first argue that it is important that health care professionals acknowledge that the health knowledge that older adults gain in the community could importantly contribute to people’s CHL. Second, for policy makers and developers of health literacy initiatives, our findings provide insight into how to strengthen the CHL of older adults as well as communities through the practices of co-learning and social support. Nevertheless, care must be taken to ensure that these efforts empower rather than disempower the access and use of health information to improve the health and well-being in communities.

## Additional files

Additional file 1: Searches in electronic databases. (DOCX 18 kb)

Additional file 2: PRISMA 2009 checklist. (DOC 62 kb)

Additional file 3: Quality assessment form used in this systematic review, based on CASP (2013) and Viswanathan et al. [21, 22]. (DOCX 18 kb)

## Abbreviations

CHL: Critical health literacy
HL: Health literacy
