# Supplementary material for: Community-based initiatives improving critical health literacy: a systematic review and meta-synthesis of qualitative evidence
Source: BMC Public Health. 2017 Jul 20;18:40. doi: 10.1186/s12889-017-4570-7 (PMC5520348; doi:10.1186/s12889-017-4570-7)
Supplement: Supplementary file 1 — Searches in electronic databases. (DOCX 18 kb) [file 12889_2017_4570_MOESM1_ESM.docx]

**Additional file 1: Searches in five electronic databases (23 December 2014)**

**Pubmed:** n=915 hits

("Community-Based Participatory Research"[Mesh] OR community based participatory[tiab] OR cbpr [tiab] OR action research[tiab] OR participatory action[tiab] OR participatory research[tiab] OR participatory approach[tiab] OR participatory intervention[tiab] OR participatory evaluation[tiab] OR participatory project[tiab] OR participatory program*[tiab])

AND

(older adults[tiab] OR elderly[tiab] OR seniors[tiab] OR older people[tiab] OR aging[tiab] OR ageing[tiab] OR aged[MeSH])

**Embase:** n=862 hits

('participatory research'/exp OR 'community based participatory':ab,ti OR cbpr:ab,ti OR 'action research':ab,ti OR 'participatory action':ab,ti OR 'participatory research':ab,ti OR 'participatory approach':ab,ti OR 'participatory intervention':ab,ti OR 'participatory evaluation':ab,ti OR 'participatory project':ab,ti OR 'participatory program':ab,ti OR 'participatory programs':ab,ti OR 'participatory programme':ab,ti OR 'participatory programmes':ab,ti)

AND

('older adults':ab,ti OR elderly:ab,ti OR seniors:ab,ti OR 'older people':ab,ti OR aging:ab,ti OR ageing:ab,ti OR 'aged'/exp)

|  | **Web of Science:** n=869 hits  ("community based participatory" OR cbpr OR "action research" OR "participatory action" OR "participatory research" OR "participatory approach" OR "participatory intervention" OR "participatory evaluation" OR "participatory project" OR "participatory program*")  AND  (aged OR "older adults" OR elderly OR seniors OR "older people" OR aging OR ageing) |
| --- | --- |

**Cinahl:** n=761 pubs

( ( TI ( "community based participatory" OR cbpr OR "action research" OR "participatory action" OR "participatory research" OR "participatory approach" OR "participatory intervention" OR "participatory evaluation" OR "participatory project" OR "participatory program*") OR AB ("community based participatory" OR cbpr OR "action research" OR "participatory action" OR "participatory research" OR "participatory approach" OR "participatory intervention" OR "participatory evaluation" OR "participatory project" OR "participatory program*") OR DE "Action Research") )

AND

( (TI ( ("older adults" OR elderly OR seniors OR "older people" OR aging OR ageing) OR AB ("older adults" OR elderly OR seniors OR "older people" OR aging OR ageing) ) )

**PsycINFO:** n=556 hits

( ( TI ( "community based participatory" OR cbpr OR "action research" OR "participatory action" OR "participatory research" OR "participatory approach" OR "participatory intervention" OR "participatory evaluation" OR "participatory project" OR "participatory program*") OR AB ("community based participatory" OR cbpr OR "action research" OR "participatory action" OR "participatory research" OR "participatory approach" OR "participatory intervention" OR "participatory evaluation" OR "participatory project" OR "participatory program*") OR DE "Action Research") )

AND

( (TI ( ("older adults" OR elderly OR seniors OR "older people" OR aging OR ageing) OR AB ("older adults" OR elderly OR seniors OR "older people" OR aging OR ageing) ) )
