# Supplementary material for: Community-based initiatives improving critical health literacy: a systematic review and meta-synthesis of qualitative evidence
Source: BMC Public Health. 2017 Jul 20;18:40. doi: 10.1186/s12889-017-4570-7 (PMC5520348; doi:10.1186/s12889-017-4570-7)
Supplement: Supplementary file 3 — Quality assessment form used in this systematic review, based on CASP (2013) and Viswanathan et al. [21, 22]. (DOCX 18 kb) [file 12889_2017_4570_MOESM3_ESM.docx]

**Additional file 3: Quality Assessment form (derived from CASP (2013) and Viswanathan et al. (2004))**

**Part 1: Aim & methodology**

1. Was there a clear statement of the aims of the research?

Choose one of the following: Yes Can’t tell No

*HINT: Consider*

*• What was the goal of the research?*

*• Why it was thought important?*

*• Its logic (problem statement -> defined objectives)*

2. Does the research use an appropriate participatory approach?

Choose one of the following: Yes Can’t tell No

*HINT: Consider*

*• Is a participatory approach the right approach for addressing the research goal?*

*• Does the research seek to engage different stakeholders in different phases of the research: from the design phase to the end*

*• Are the research outcomes translated into interventions/actions*

3. Does the research use an appropriate qualitative methodology ?

Choose one of the following: Yes Can’t tell No

*HINT: Consider*

*• Is qualitative research the right methodology for addressing the research goal?*

*• Does the research seek to interpret or illuminate the actions and/or subjective experiences of research participants?*

4. Is the research about CHL?

Choose one of the following: Yes Can’t tell No

*HINT: Consider*

*• Is the study about HL, defined as “understanding and ability to judge, sift and use information provided in the context of one’s own life” (Kickbusch, 2001)?*

*• Are informed health-decisions and actions addressed?*

*• Are people studied in their cultural, social, economic and political context?*

*• Are all determinants of health and wellbeing at the individual as well as the population level of society taken into account?*

**Part 2: Community-Based Participatory Elements**

1. Nature of Community Involvement

1a. Selection of research question

1b. Proposal development

1c. Financial responsibility for grant funds

1d. Study design

1e. Recruitment and retention of study participants

1f. Measurement instruments and data collection

1g. Intervention development, implementation

1h. Interpretation of findings

1i. Dissemination of findings

1j. Application of findings to health concern identified

2. Evidence of Community-Based Participatory Research Elements:

2a. Structure or mechanism for shared decision-making between researchers and the

community

2b. Study was designed to remove barriers to community participation in research

2c. Socio-economic determinants of health were:

2ci Assessed through design of the study or intervention

2cii Addressed through design of the study or intervention

2d. Research team was flexible to community needs and priorities during research

implementation

2e. Study’s duration and purpose contributed to:

2ei Individual capacity building

2eii Community capacity building

2f. Findings were either used or intended to be used to address the original health concerns with

regard to:

2fi. Dissemination to participants

2fii. Application to a health related intervention or policy change

2fiii. Sustainability of research-related interventions in the community

*Rating scheme:*

3 = Good

2 = Fair

1p = Poor

1in = Insufficient information reported to determine

NA = Not Applicable
